# Cholesterol and the risk of grade-specific prostate cancer incidence: evidence from two large prospective cohort studies with up to 37 years' follow up

**DOI:** 10.1186/1471-2407-12-25

**Published:** 2012-01-19

**Authors:** Kashif Shafique, Philip McLoone, Khaver Qureshi, Hing Leung, Carole Hart, David S Morrison

**Affiliations:** 1 Institute of Health & Wellbeing, Public Health, University of Glasgow, 1 Lilybank Gardens, Glasgow G12 8RZ, UK; 2 West of Scotland Cancer Surveillance Unit, University of Glasgow, 1 Lilybank Gardens, Glasgow G12 8RZ, UK; 3 Urology Department, Gartnavel General Hospital, 1053 Great Western Road, Glasgow G12 0YN, UK; 4 Beatson Institute for Cancer Research, Garscube Estate, Switchback Road, Bearsden, Glasgow G61 1BD, UK

**Keywords:** Cholesterol, Prostate cancer, Incidence, Gleason grade

## Abstract

**Background:**

High cholesterol may be a modifiable risk factor for prostate cancer but results have been inconsistent and subject to potential "reverse causality" where undetected disease modifies cholesterol prior to diagnosis.

**Methods:**

We conducted a prospective cohort study of 12,926 men who were enrolled in the Midspan studies between 1970 and 1976 and followed up to 31st December 2007. We used Cox-Proportional Hazards Models to evaluate the association between baseline plasma cholesterol and Gleason grade-specific prostate cancer incidence. We excluded cancers detected within at least 5 years of cholesterol assay.

**Results:**

650 men developed prostate cancer in up to 37 years' follow-up. Baseline plasma cholesterol was positively associated with hazard of high grade (Gleason score≥8) prostate cancer incidence (n = 119). The association was greatest among men in the 2nd highest quintile for cholesterol, 6.1 to < 6.69 mmol/l, Hazard Ratio 2.28, 95% CI 1.27 to 4.10, compared with the baseline of < 5.05 mmol/l. This association remained significant after adjustment for body mass index, smoking and socioeconomic status.

**Conclusions:**

Men with higher cholesterol are at greater risk of developing high-grade prostate cancer but not overall risk of prostate cancer. Interventions to minimise metabolic risk factors may have a role in reducing incidence of aggressive prostate cancer.

## Background

The incidence of prostate cancer has increased over several decades such that it is now the most commonly diagnosed cancer among men in Europe and the USA [1,2]. There is evidence that increasing age, genetic predisposition [3,4] and ethnicity [5] are risk factors for prostate cancer while opportunistic testing may partly explain temporal increases and international variations in incidence [1]. Prostate cancer is about six times more common in more developed compared to less developed countries, suggesting that modifiable Western lifestyle factors may have a causal role [6]. It has been observed for about a century that the levels of cholesterol, fatty deposits, lecithin and some other lipids in the diseased prostate are elevated [7]. Several studies have explored the relationship between serum cholesterol levels and the incidence of prostate cancer and its associated mortality with inconsistent conclusions [8-13]. Some found a positive association between cholesterol and prostate cancer mortality [8,14] while others revealed either an inverse relationship [10,13] or no overall association with incidence [9,11].

Three recent reports have suggested that while serum cholesterol has no association with overall incidence of prostate cancer, patients with low cholesterol are less likely to have high grade (Gleason score ≥8) disease [15-17]. In two of these studies, follow-up was short (3.1 and 5.5 years) [16,17] and none excluded early events so that "reverse causality"--that is, cholesterol was modified by undiagnosed disease and not a causal factor for it--may have partly explained their observations.

Given that age, genetics and ethnicity are not modifiable risk factors, the potential role of cholesterol on prostate cancer risk may be of clinical importance. In the present study we evaluated the association between plasma cholesterol level and both overall and grade-specific prostate cancer incidence, using two of the Midspan prospective cohort studies with up to 37 years' follow-up. Individuals diagnosed with prostate cancer within 5 years of baseline cholesterol assay were excluded to reduce the potential effects of "reverse causality."

## Methods

### Cohort characteristics

The Midspan studies began in the 1960s and 1970s in Scotland, UK. Their methods have been described in detail elsewhere [18]. We included two Midspan studies in this research paper. The first, the Collaborative study, was conducted on employed men and women aged from 21 to 75 years from 27 workplaces in Glasgow, Clydebank and Grangemouth between 1970 and 1973 [19]. The second Midspan study, the Renfrew/Paisley study, was a general population study of residents of the towns of Renfrew and Paisley, conducted between 1972 and 1976. All residents aged 45-64 years were invited to take part and 80% accepted [20].

Because of the geographical proximity of the study populations, a small number of individuals participated in both cohorts. For individuals with more than one record, only the earliest record was used. Study protocols consisted of a self-administered questionnaire followed by a screening examination at a specially set-up clinic. Questions included demographic details, occupation, lifestyle habits, including smoking, and health [20]. As part of the screening examination, measurements were made for height and weight and blood pressure. A blood sample was obtained at baseline screening for the measurement of total circulating plasma cholesterol. Body mass index (BMI) was calculated from weight (in kg) divided by height (in metres) squared and categorised according to the World Health Organisation [21] classification in which BMI < 18.50 is underweight, 18.50 to 24.99 is the normal range, 25.00 to 29.99 is overweight and ≥30.00 is obese. A blood sample was obtained at baseline screening for the measurement of total circulating plasma cholesterol [20]. Socioeconomic status was derived from occupation according to the relevant version of the General Register Office Classification of Occupation [22] and graded into six categories: I [23], II (intermediate), III (skilled non-manual), III (skilled manual), IV (partly skilled) and V (unskilled) [20]. Ex-smokers were defined as reporting giving up smoking at least a year before screening, otherwise they were defined as current smokers. Cholesterol was categorised by quintiles. Only records for male participants were used for this study.

### Follow up

Follow up for mortality was carried out by flagging Midspan participants with the National Health Service Central Register. Deaths were then notified to the Midspan team on a monthly basis. Information on cancer registrations and hospital activity was obtained by linkage to the Scottish Morbidity Records (SMR) data and was complete from 1972 onwards [24]. Follow up began on the date of screening to the date of cancer incidence, date of death, date of embarkation (leaving the United Kingdom) or the censor date of 31st December 2007, whichever came first.

### Ethical approval

The Privacy Advisory Committee of the Information Services Division of NHS Scotland gave permission for the linked data to be used in this study.

### Risk factor and outcome definitions

Prostate cancer was defined as International Classification of Diseases (ICD) revision 9 codes 185 and ICD-10 codes C61. Prostate cancer incidence was determined if it was included in any cancer registration (SMR06), any diagnosis position of an acute hospital record (SMR01) or any position on the death record. Where a patient had prostate cancer recorded on more than one type of record, the earliest date was taken as time of first diagnosis. The Gleason grading system is a method used to describe the morphology of clinical PC. Data on Gleason score were available from the cancer registry (SMR06). The Scottish Cancer Registry began recording Gleason score from 1st January 1997 and therefore the analysis of grade-specific associations between cholesterol and PC was restricted to the follow up of the surviving cohort as of 1st of January 1997 and these were just records from SMR06.

### Statistical analysis

Cox proportional hazards models were used to estimate hazard ratios (HRs) for PC incidence from screening and for specific histological grade from 1st January 1997. For grade specific analysis, we excluded men who had died or been diagnosed with PC before 1^st ^January 1997. Separate models were run for each Gleason category, and men with PC and other Gleason scores were censored at their date of diagnosis. Age was taken as the timescale with censoring occurring on 31 December 2007. The alternative approach of using time since screening as the timescale was also investigated [25]. The estimates presented in this study are obtained by using age as the time scale. All analyses were conducted using STATA version 11 (StataCorp, College Station, TX, USA). The following covariates were included in the multivariate model: smoking status, BMI and socioeconomic status and results are presented based on this model. There is some evidence that increasing height significantly increases the risk of developing PC, so BMI was replaced with height in the second model and estimates for height are presented when BMI was not included in the model. There were missing data for some covariates: 89 in socioeconomic status; 1 in height and weight; 1 in smoking status; Total missing data for all covariates was less than 0.01% which did not change any of the associations when we ran the analysis both including those observations after imputations and excluding these individuals. We presented the final results after imputations in which missing information on continuous variables was replaced by the sample mean while for categorical variables missing data were replaced by modal values. The lowest category was used as referent for the cholesterol and all other categorical covariates. The analysis was repeated by combining the two highest quintiles of cholesterol. Furthermore, analysis was also carried out by using the recommended clinical cut offs for adults cholesterol level, where cholesterol level of less than 5.1 mmol/L was considered as desirable, 5.1 to < 6.21 mmol/L as borderline high and ≥6.21 mmol/L as high [26]. Analysis was also stratified based on BMI categories (i.e. desirable, overweight, obese), and also by using the median BMI of the sample, consistent with an earlier study [15]. Adherence to the proportional hazards assumption was investigated by plotting smoothed Schoenfeld residuals against time; no violations of the assumption were identified. All statistical tests were two tailed and statistical significance was taken as *p *< 0.05. The analysis was carried out after excluding individuals diagnosed with PC within 5 years of screening to minimise confounding due to the possible effects of early disease affecting cholesterol [27].

## Results

Data from 13,071 men were available for analysis, 6022 (46.1%) from the Collaborative Study and 7049 (53.9%) from Renfrew/Paisley. Five Collaborative and nine Renfrew/Paisley participants were lost to follow up, and 42 Collaborative and 55 Renfrew/Paisley participants had missing cholesterol data. Twenty six individuals who participated in both the studies were excluded from the Renfrew/Paisley study. Eight individuals diagnosed with PC in the first 5 years of screening were also excluded from the analysis. Our final sample therefore comprised 12,926 men followed-up for a total of 293,284 person-years. The median follow-up period was 24 years, maximum 37 years. Median age was 51 years at the time of screening (range, 21-75 years).

Baseline and outcome characteristics for the study are shown in Table 1. Six hundred and fifty men with PC were identified. Among 307 cancers that occurred from 1997 onward (when Gleason score was included in cancer registry data), 119 (38.8%) were high grade (Gleason score≥8), 57 (18.6%) were intermediate grade (Gleason = 7), 64 (20.8%) low grade (Gleason≤6) and the remaining 67 (21.8%) were of unknown Gleason score. Increasing weight and BMI were positively associated with cholesterol while current smoking and lower socioeconomic status were inversely associated with cholesterol (data not shown). Mean plasma cholesterol level did not differ (*p *= 0.27) between men who were diagnosed with prostate cancer (5.85 mmol/l ± 0.99) and those who remained free from it (5.87 mmol/l ± 0.99). The mean time between screening (plasma cholesterol measurement) and the prostate cancer diagnosis was 22.9 (SD 7.84) years.

**Table 1:** Baseline characteristics of male Midspan cohorts at screening (1970-76) and prostate cancer outcomes. N = 12,926

	Controls		Prostate cancer cases		Gleason < 7		Gleason = 7		Gleason 8-10		Unknown Gleason	
Sample Size	12,276		650		64		57		119		67	
*Number of Deaths%(n)*	76.4	(9,384)	81.1	(527)	51.6	(33)	52.6	(30)	73.9	(88)	82.1	(55)
*Cholesterol quintiles, mmol/L,%(n)*												
*< 5.05*	94.9	(2,589)	5.1	(138)	24.6	(15)	21.3	(13)	27.9	(17)	26.2	(16)
*5.06 - < 5.57*	95.3	(2,471)	4.7	(121)	17.7	(9)	13.7	(7)	37.2	(19)	31.4	(16)
*5.58 - < 6.09*	94.9	(2,676)	5.1	(145)	26.1	(17)	12.3	(8)	46.2	(30)	15.4	(10)
*6.1 - < 6.69*	94.5	(2,161)	5.5	(125)	16.9	(12)	18.3	(13)	46.5	(33)	18.3	(13)
*≥6.7*	95.2	(2,379)	4.8	(121)	18.6	(11)	27.1	(16)	33.9	(20)	20.3	(12)
Mean Age (s.d.)	51.1	(7.2)	51.7	(6.8)	46.9	(6.8)	46.7	(6.3)	49.2	(6.3)	50.7	(6.5)
Mean Height (s.d.)	171.1	(7.1)	172.0	(6.9)	172.6	(7.3)	173.3	(6.1)	172.3	(6.5)	172.6	(7.3)
Mean Weight (s.d.)	74.8	(11.1)	75.9	(10.4)	75.7	(10.8)	76.0	(9.0)	74.9	(9.5)	76.3	(9.6)
*Height (cm),%(n)*												
≤165.1	95.9	(2,770)	4.1	(120)	24.5	(12)	14.3	(7)	34.7	(17)	26.5	(13)
165.2-170	95.3	(2,551)	4.7	(126)	19.1	(9)	12.8	(6)	49.0	(23)	19.1	(9)
170.1-172.72	95.0	(2,321)	5.0	(121)	19.7	(13)	27.3	(18)	33.3	(22)	19.7	(13)
172.73-177.8	94.3	(2,745)	5.7	(165)	19.0	(16)	17.9	(15)	42.9	(36)	20.2	(17)
≥177.9	94.1	(1,887)	5.9	(118)	23.0	(14)	18.0	(11)	34.4	(21)	24.6	(15)
Missing	0.0	(2)	0.0	(0)	0.0	(0)	0.0	(0)	0.0	(0)	0.0	(0)
Mean BMI (s.d.)	25.5	(3.3)	25.6	(3.0)	25.4	(3.0)	25.3	(2.7)	25.2	(3.0)	25.6	(3.0)
*BMI (kg m^-2^),%(n)*												
< 25 (Under & Desirable weight )	95.2	(5,499)	4.8	(279)	20.8	(31)	17.5	(26)	40.9	(61)	20.8	(31)
25 - < 30 (Overweight)	94.7	(5,729)	5.3	(320)	21.9	(30)	19.7	(27)	34.3	(47)	24.1	(33)
≥30 (Obese)	95.4	(1,046)	4.6	(51)	14.3	(3)	19.0	(4)	52.4	(11)	14.3	(3)
Missing	0.0	(2)	0.0	(0)	0.0	(0)	0.0	(0)	0.0	(0)	0.0	(0)
*Smoking,%(n)*												
Never smoker	93.3	(2,093)	6.7	(149)	22.8	(18)	19.0	(15)	39.2	(31)	19.0	(15)
Smoker	96.1	(7,253)	3.9	(294)	18.3	(24)	17.6	(23)	39.7	(52)	24.4	(32)
Ex-smoker	93.4	(2,929)	6.6	(207)	22.7	(22)	19.6	(19)	37.1	(36)	20.6	(20)
Missing	0.0	(1)	0.0	(0)	0.0	(0)	0.0	(0)	0.0	(0)	0.0	(0)
*Social Class,%(n)*												
I&II	94.2	(3,090)	5.8	(190)	16.3	(16)	23.5	(23)	38.8	(38)	21.4	(21)
IIIN	94.4	(1,810)	5.6	(108)	27.8	(15)	11.1	(6)	40.7	(22)	20.4	(11)
IIIM	95.3	(4,247)	4.7	(208)	18.1	(15)	19.3	(16)	37.3	(31)	25.3	(21)
IV&V	95.8	(3,057)	4.2	(135)	25.0	(17)	16.2	(11)	38.2	(26)	20.6	(14)
Missing	88.9	(72)	11.1	(9)	25.0	(1)	25.0	(1)	50.0	(2)	0.0	(0)

**Figure 1:**
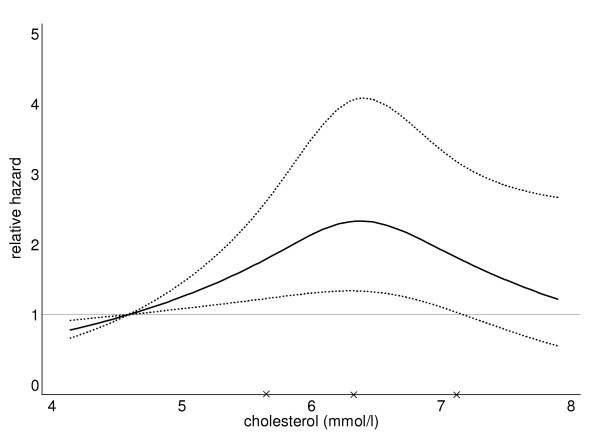
**Functional form of the association of cholesterol with the relative hazard of Gleason 8 to 10 prostate cancers estimated in a Cox proportional hazards model using age as the time axis**. The function was fitted using restricted cubic splines with three knots (X). The function was standardized such that the HR was 1 at the mean cholesterol level of the lowest quintile. Dotted lines indicate the 95% confidence intervals.

Using age as the time-scale we found no convincing association between cholesterol and overall hazard of PC, nor any consistent relationship within low and intermediate grade disease (Table 2). However, the hazard increased consistently from the lowest to the second highest quintile of cholesterol among high grade disease (Gleason score ≥ 8). We explored the relationship between cholesterol and high grade disease further in Table 3. After adjustment for BMI, smoking and socioeconomic status, a progressive increase in risk of high grade prostate remained between the lowest and second highest quintiles of cholesterol. This is more clearly shown in Figure 1, in which the smoothed hazard of the most aggressive PCs (Gleason score≥8) increased with increasing cholesterol and then declined. As no significant association was observed between the highest quintile of cholesterol and risk of high grade disease, we combined the last two quintiles to further investigate the association. We observed significantly higher risk (HR 1.88, 95 CI 1.08-3.27, p-value 0.03) of developing high grade disease among men in the highest cholesterol category (combination of 4^th ^and 5^th ^quintile). Furthermore, we also investigated the association between cholesterol and risk of high grade disease using the clinical cut points. Men in the higher cholesterol group (≥ 6.21 mmols/l) had significantly increased risk of developing high grade disease (1.75, 95% CI 1.03-2.97, p value 0.036) compared to the desirable cholesterol group (< 5.1 mmols/l) after adjustment for BMI, smoking and socioeconomic status (data not shown).

**Table 2:** Unadjusted hazard ratios and 95% CI for overall and Gleason-specific prostate cancer by cholesterol quintiles among Midspan subjects who survived until 1^st ^January 1997. N = 6486.

		All Prostate cancer				Gleason < 7				Gleason 7				Gleason ≥8				Unknown			
***Cholesterol (mmol/L) quintiles***			**(n)**	**Hazard Ratio (95%CI)**		%	**(n)**	**Hazard Ratio (95%CI)**		%	**(n)**	**Hazard Ratio (95%CI)**		%	**(n)**	**Hazard Ratio (95%CI)**		%	**(n)**	**Hazard Ratio (95%CI)**	

< 5.05		1,384	(61)		1	24.6	(15)		1	21.3	(13)		1	27.9	(17)		1	26.2	(16)		1
5.06 - < 5.57		1,308	(51)	0.87	(0.60, 1.26)	17.6	(9)	0.62	(0.27,1.41)	13.7	(7)	0.54	(0.22,1.36)	37.3	(19)	1.18	(0.61, 2.27)	31.4	(16)	1.04	(0.52, 2.09)
5.58 - < 6.09		1,425	(65)	1.03	(0.72, 1.46)	26.2	(17)	1.1	(0.56,2.27)	12.3	(8)	0.59	(0.24,1.42)	26.2	(30)	1.70	(0.93, 3.07)	15.4	(10)	0.59	(0.27, 1.31)
6.1 - < 6.69		1,159	(71)	1.36	(0.97, 1.92)	16.9	(12)	0.96	(0.45,2.05)	18.3	(13)	1.14	(0.53,2.47)	46.5	(33)	2.28	(1.27, 4.10)	18.3	(13)	0.95	(0.46, 1.98)
≥6.7		1,210	(59)	1.09	(0.76, 1.57)	18.6	(11)	0.83	(0.38,1.81)	27.1	(16)	1.35	(0.65,2.81)	33.9	(20)	1.34	(0.70, 2.57)	20.3	(12)	0.85	(0.40, 1.80)

Univariate hazard ratios and 95% confidence intervals were estimated within a model employing age as time-scale

**Table 3:** Multivariate hazard ratios (HR) for all prostate cancers and those with Gleason grade ≥ 8 by cholesterol quintiles

	All Prostate Cancers					Prostate cancers Gleason ≥ 8				
	**Baseline Sample**					**From 1st January 1997**				

	**%**	**n**	**Hazard Ratio (95% CI)**			**%**	**n**	**Hazard Ratio (95% CI)**		

*Cholesterol (mmol/L) quintiles*										
< 5.05	5.1	(138)		1		1.2	(17)		1	
5.06 - < 5.57	4.7	(121)	0.89	(0.70, 1.14)		1.5	(19)	1.18	(0.62, 2.28)	
5.58 - < 6.09	5.1	(145)	0.95	(0.75, 1.21)		2.1	(30)	1.72	(0.95, 3.13)	
6.1 - < 6.69	5.5	(125)	1.01	(0.79, 1.29)		2.8	(33)	2.34	(1.30, 4.23)	
≥6.7	4.8	(121)	0.95	(0.74, 1.22)		1.7	(20)	1.40	(0.73, 2.71)	
*BMI (kg m^-2^)*										
< 25 (Under & Desirable weight)	4.8	(279)		1		2.1	(61)		1	
25 - < 30 (Overweight)	5.3	(320)	1.02	(0.86, 1.20)		1.5	(47)	0.69	(0.47, 1.02)	
≥30 (Obese)	4.6	(51)	1.03	(0.77, 1.40)		2.3	(11)	1.18	(0.62, 2.27)	
*Smoking*										
Never smoker	6.6	(149)		1		2.1	(31)		1	
Smoker	3.9	(294)	0.90	(0.73, 1.09)		1.6	(52)	0.92	(0.59, 1.45)	
Ex-smoker	6.6	(207)	1.03	(0.77, 1.40)		2.1	(36)	1.06	(0.65, 1.72)	
*Social Class*										
I&II	5.8	(190)		1		1.9	(38)		1	
IIIN	5.6	(108)	1.10	(0.87, 1.40)		2.2	(22)	1.21	(0.71, 2.05)	
IIIM	4.7	(217)	1.03	(0.85, 1.26)		1.6	(33)	0.95	(0.59, 1.53)	
IV&V	4.2	(135)	0.91	(0.73, 1.14)		1.9	(26)	1.15	(0.69, 1.92)	
*Height (cm)*										
≤165.1	4.9	(120)		1		1.8	(17)		1	
165.2-170	4.7	(126)	1.05	(0.82, 1.35)		1.8	(23)	1.27	1(0.68, 2.37)	
170.1-172.72	4.2	(121)	1.11	(0.86, 1.43)		1.4	(22)	1.54	(0.61, 2.19)	
172.73-177.8	7.5	(165)	1.27	(1.01, 1.61)		3.2	(36)	1.69	(0.94, 3.05)	
≥177.9	4.3	(118)	1.35,	(1.04 1.75)		1.3	(21)	1.32	(0.68, 2.55)	

Multivariate hazard ratios and 95% CIs were obtained by using age as time-scale; all covariates were included in the model except height. Estimates for height were obtained by replacing the BMI with height

We further stratified the analysis based on BMI. The association between cholesterol level and high grade prostate cancer differed by BMI, however, no clear relationship emerged when using the desirable, overweight and obese categories, due to smaller number of aggressive PC cases in two highest cholesterol quintiles of obese group (n = 7), so analysis was then stratified based on the median BMI of the sample. There was no evidence of an association between cholesterol level and risk of high grade disease in men with BMIs lower than 25.3. However, among men with high BMI (≥ 25.3, median of the sample), those in the second highest cholesterol quintile were significantly more likely to develop high grade disease (HR 9.98, 95% CI 2.33-42.78, p value 0.002) after adjustments for socioeconomic status and smoking status (data not shown). Similarly among men with higher BMI, when we combined the two highest cholesterol quintiles, those in the highest category had seven times higher risk of developing high grade disease compared to the lowest cholesterol group. We also examined the interaction between cholesterol and BMI within multivariate model, however, no significant interaction was observed between BMI and cholesterol (p for interaction 0.86). We also noted a progressive increase in risk of all PCs with increasing height in univariate analysis, for this reason we also ran a multivariate model using height instead of BMI to examine any confounding effect, however, the associations of cholesterol and height with PC incidence remained consistent after adjusting for smoking and socioeconomic status (Table 3).

## Discussion

We found that plasma cholesterol was positively associated with increased risk of aggressive prostate cancer but not with overall risk of developing the disease in this population-based prospective cohort study. Our findings are consistent with others reported on United States populations [15-17]. Similarly, data from the Swedish Apolipoprotein Mortality Risk (AMORIS) study reported no evidence of a relationship between hypercholesterolemia and overall prostate cancer risk [28]. However, a large study of male Finnish smokers, reported a positive association between increasing total cholesterol level and overall risk of prostate cancer particularly advanced stage prostate cancer [29]. The association further strengthened when they restricted the analysis to the cases diagnosed after 10 years from the baseline. However, this study did not find any association between aggressive disease, which may be because Gleason score was only available for 25% of the prostate cancer patients [29].

Several underlying mechanisms by which cholesterol and prostate carcinogenesis may be linked have been proposed. Prostate cancer cells tend to over accumulate cholesterol in their cell membrane, forming large lipid rafts which in the cancer cells may facilitate pro-carcinogenic cell signalling [29-31]. Moreover, several other pathways which are considered vital in carcinogenesis, such as sonic hedgehog and Akt pathways, are also cholesterol sensitive [32,33]. Thus, having a lower cholesterol level may inhibit these pro-carcinogenic activities in the prostate cells.

Generally, an association has been reported between low cholesterol and increased risk of many cancer types and their associated mortality [9,11,12] which has been ascribed to reverse causality; that is, early undiagnosed cancers lead to behavioural and physiological changes that reduce plasma cholesterol. The longer period between baseline cholesterol assay and diagnosis in our study (about 21 years for grade-specific analyses) compared to others suggests that reverse causality is unlikely to have been responsible for the observed association. Moreover, any such effect would have been expected to attenuate rather than exaggerate the association.

The potential clinical implications of our findings are that increasing obesity and associated dyslipidemia may have been responsible for the increasing incidence of prostate cancer and that modifying cholesterol may reduce incidence of more aggressive disease. The evidence that statins may reduce prostate cancer incidence remains equivocal. Two meta-analyses and a subsequent cohort study did not find any relationship between statin use and prostate cancer [34-36]. However, Platz and Jacobs found associations between statin use and lower risk of advanced prostate cancer only and suggested that plausible biological mechanisms may existed, for example 3-hydroxy-3-methylglutaryl (HMG) coenzyme A reductase inhibition may reduce prostate cancer cell survival by interfering with membrane-associated signalling. In the absence of more consistent evidence on the effects of statins on prostate cancer, the most effective means of reducing incidence of the disease may therefore be through effective weight management.

Our research is based on one of the largest population based prospective studies in the United Kingdom and used cancer incidence data for the grade-specific analyses, rather than death records for cancer outcomes. Mortality data are a product of both incidence and case fatality, and do not allow risk factors to be individually differentiated. Furthermore, high cholesterol may increase the risk of death from other causes in prostate cancer patients and not necessarily be a causal factor for prostate cancer itself. Our study has larger numbers of incident cancers (n = 650), longer follow up and lower losses to follow up (0.1%) compared with earlier studies [15,16]. However, our study has some weaknesses. The number of cases with aggressive PC was smaller; this may have influenced our results in some analyses. The Midspan questionnaire lacked information on family history of PC, prostate specific antigen screening and use of statins. We used plasma total cholesterol level because other measures of cholesterol, such as lipoprotein fractions (high and low density lipoproteins), were not available. Prostate specific antigen and disease stage data were not available which could be used to stratify the analyses based on localised and metastatic prostate cancer. The mean age at prostate cancer diagnosis is high and a large proportion of men are likely to die before diagnosis. The risk estimates we present might therefore have been affected by differential competing mortality risks. However, Cancer Registry data include Death Certificate Only diagnoses--and may have included, prostate cancers detected at post-mortem--that will attenuate such survival biases. The proportion of men who develop PC is higher among those who do not smoke, have a desirable BMI and are taller. The higher proportion of PC among these men results from those factors which confer a survival advantage. They live longer and therefore experience a longer risk time. However, the observation that height is associated with PC does raise the question whether some of those characteristics which promote longevity, are also associated with an increased risk of PC or whether such associations spuriously result from the influence of competing risk. Height is linked with development of many adult cancers [37-39], a meta-analysis of 58 studies also suggested that height is positively associated with incident prostate cancer with stronger effect for advance stage and aggressive disease [40]. There is some suggestion that height might be confounded by the socioeconomic status of the individual, however in our study height was associated with overall risk of prostate cancer independent of socioeconomic status.

Cholesterol, height and obesity are related to mortality from cardiovascular disease [27,41-43] and early death from cardiovascular disease may be an important consideration. One possible scenario is that early cardiovascular disease mortality exhausts the pool of those men who would otherwise be susceptible to PC in later life, consequently systematic selection of more resilient individual may take place (men with low risk of PC but with high levels of traditional cardiovascular risk factors). This potentially could explain the positive association between height and overall incident PC, but would fail to explain the association between cholesterol and aggressive PC, which we report. A further consideration is detection bias and whether some groups are more likely to report their symptoms or have frequent medical examinations.

Further research is needed on cancer registry data to determine whether high-grade prostate cancer has differentially increased following increases in metabolic factors associated with hypercholesterolaemia. Longer-term follow-up of clinical trials of statins is also required, with exclusions of early tumours to minimise the potential effects of "reverse causality" on any association.

## Conclusion

In this population-based cohort study, high cholesterol level was associated with increased risk of aggressive prostate cancer; these findings support the results from earlier studies. Further research is needed to describe temporal trends in grade-specific prostate cancer and to understand the biological mechanisms by which cholesterol and prostate cancer are associated.

## Competing interests

The authors declare that they have no competing interests.

## Authors' contributions

All authors designed the study; KS and PM carried out statistical analyses; all authors contributed to interpreting the results; KS, PM and DSM drafted the manuscript; all authors saw and approved the final manuscript.

## Pre-publication history

The pre-publication history for this paper can be accessed here:

http://www.biomedcentral.com/1471-2407/12/25/prepub

## References

[B1] Bray F Lortet-Tieulent J Ferlay J Forman D Auvinen A Prostate cancer incidence and mortality trends in 37 European countries: an overview Eur J Cancer 2010 46 3040 3052 10.1016/j.ejca.2010.09.013 21047585

[B2] Jemal A Siegel R Ward E Hao Y Xu J Murray T Cancer statistics, 2008 CA Cancer J Clin 2008 58 71 96 10.3322/CA.2007.0010 18287387

[B3] Johns LE Houlston RS A systematic review and meta-analysis of familial prostate cancer risk BJU Int 2003 91 789 794 10.1046/j.1464-410X.2003.04232.x 12780833

[B4] Sakr WA Grignon DJ Haas GP Heilbrun LK Pontes JE Crissman JD Age and racial distribution of prostatic intraepithelial neoplasia Eur Urol 1996 30 138 144 8875194 10.1159/000474163

[B5] Ben-Shlomo Y Evans S Ibrahim F Patel B Anson K Chinegwundoh F The risk of prostate cancer amongst black men in the United Kingdom: the PROCESS cohort study Eur Urol 2008 53 99 105 10.1016/j.eururo.2007.02.047 17368710

[B6] Baade PD Youlden DR Krnjacki LJ International epidemiology of prostate cancer: geographical distribution and secular trends Mol Nutr Food Res 2009 53 171 184 10.1002/mnfr.200700511 19101947

[B7] White RM On the occurrence of crystals in tumours J Pathol Bacteriol 1909 13 3 10 10.1002/path.1700130103

[B8] Bravi F Scotti L Bosetti C Talamini R Negri E Montella M Self-reported history of hypercholesterolaemia and gallstones and the risk of prostate cancer Ann of Oncol 2006 17 1014 1017 10.1093/annonc/mdl080 16611646

[B9] Hiatt RA Fireman BH Serum cholesterol and the incidence of cancer in a large cohort J Chr Dis 1986 39 861 870 10.1016/0021-9681(86)90034-2 3793838

[B10] Kark JD Smith AH Hames CG Serum retinol and the inverse relationship between serum cholesterol and cancer Br Med J 1982 284 152 154 10.1136/bmj.284.6310.152 PMC1495538 6799076

[B11] Knekt P Reunanen A Aromaa A Heliovaara M Hakulinen T Hakama M Serum cholesterol and risk of cancer in a cohort of 39,000 men and women J Clin Epidemiol 1988 41 519 530 10.1016/0895-4356(88)90056-X 3290396

[B12] Davey Smith G Shipley MJ Marmot MG Rose G Plasma cholesterol concentration and mortality. The Whitehall Study JAMA 1992 267 70 76 10.1001/jama.1992.03480010078028 1727199

[B13] Thompson MM Garland C Barrett-Connor E Khaw K-T Friedlander NJ Wingard DL Heart disease risk factors, diabetes, and prostatic cancer in an adult community Am J Epidemiol 1989 129 511 517 2916544 10.1093/oxfordjournals.aje.a115162

[B14] Batty GD Kivimaki M Clarke R Davey Smith G Shipley MJ Modifiable risk factors for prostate cancer mortality in London: forty years of follow-up in the Whitehall study Canc Causes Contr 2011 22 311 318 10.1007/s10552-010-9691-6 PMC3226949 21116843

[B15] Mondul AM Clipp SL Helzlsouer KJ Platz EA Association between plasma total cholesterol concentration and incident prostate cancer in the CLUE II cohort Canc Causes Contr 2010 21 61 68 10.1007/s10552-009-9434-8 PMC3004752 19806465

[B16] Platz EA Clinton SK Giovannucci E Association between plasma cholesterol and prostate cancer in the PSA era Int J Cancer 2008 123 1693 1698 10.1002/ijc.23715 18646186 PMC2536746

[B17] Platz EA Till C Goodman PJ Parnes HL Figg WD Albanes D Men with low serum cholesterol have a lower risk of high-grade prostate cancer in the placebo arm of the prostate cancer prevention trial Cancer Epidemiol Biomarkers Prev 2009 18 2807 2813 10.1158/1055-9965.EPI-09-0472 19887582 PMC2877916

[B18] Hart CL MacKinnon PL Watt GC Upton MN McConnachie A Hole DJ The Midspan studies Int J Epidemiol 2005 34 28 34 15705739 10.1093/ije/dyh348

[B19] Davey Smith G Hart C Hole D MacKinnon P Gillis C Watt G Education and occupational social class: Which is the more important indicator of mortality risk? J Epidemiol Community Health 1998 52 153 160 10.1136/jech.52.3.153 9616419 PMC1756692

[B20] Hawthorne VM Watt GC Hart CL Hole DJ Davey Smith G Gillis CR Cardiorespiratory disease in men and women in urban Scotland: baseline characteristics of the Renfrew/Paisley (midspan) study population Scott Med J 1995 40 102 107 8787108 10.1177/003693309504000402

[B21] Resnick MJ Canter DJ Guzzo TJ Brucker BM Bergey M Sonnad SS Does race affect postoperative outcomes in patients with low-risk prostate cancer who undergo radical prostatectomy? Urology 2009 73 620 623 10.1016/j.urology.2008.09.035 19100607

[B22] General Register Office Classification of Occupation 1966 London: HMSO 1 136

[B23] Giovannucci E Liu Y Platz EA Stampfer MJ Willett WC Risk factors for prostate cancer incidence and progression in the health professionals follow-up study Int J Cancer 2007 121 1571 1578 10.1002/ijc.22788 17450530 PMC2430098

[B24] Hart CL Batty GD Morrison DS Mitchell RJ Davey Smith G Obesity, overweight and liver disease in the Midspan prospective cohort studies Int J Obesity 2010 34 1051 1059 10.1038/ijo.2010.20 PMC2887083 20142829

[B25] Korn EL Graubard BI Midthune D Time-to-event analysis of longitudinal follow-up of a survey: choice of the time-scale Am J Epidemiol 1997 145 72 80 8982025 10.1093/oxfordjournals.aje.a009034

[B26] National Heart, Lung and Blood Institute Third Report of the National Cholesterol Education Program (NCEP) Expert Panel on Detection, Evaluation, and Treatment of High Blood Cholesterol in Adults (Adult Treatment Panel III) Bethesda, MD: National Heart, Lung, and Blood Institute of Health 2002 NIH publication 02-5215 accessed on 29^th ^Nov, 2011

[B27] Lawlor DA Hart CL Hole DJ Davey Smith G Reverse causality and confounding and the associations of overweight and obesity with mortality Obesity (Silver Spring) 2006 14 2294 2304 10.1038/oby.2006.269 17189558

[B28] Van HM Garmo H Holmberg L Walldius G Jungner I Hammar N Prostate cancer risk in the Swedish AMORIS study: the interplay among triglycerides, total cholesterol, and glucose Cancer 2011 117 2086 95 10.1002/cncr.25758 21523720

[B29] Mondul AM Weinstein SJ Virtamo J Albanes D Serum total and HDL cholesterol and risk of prostate cancer Canc Causes Contr 2011 22 1545 1552 10.1007/s10552-011-9831-7 PMC3500884 21915616

[B30] Freeman MR Solomon KR Cholesterol and prostate cancer J Cell Biochem 2004 91 54 69 10.1002/jcb.10724 14689582

[B31] Hager MH Solomon KR Freeman MR The role of cholesterol in prostate cancer Curr Opin Clin Nutr Metab Care 2006 9 379 385 10.1097/01.mco.0000232896.66791.62 16778565

[B32] Oh HY Lee EJ Yoon S Chung BH Cho KS Hong SJ Cholesterol level of lipid raft microdomains regulates apoptotic cell death in prostate cancer cells through EGFR-mediated Akt and ERK signal transduction Prostate 2007 67 1061 1069 10.1002/pros.20593 17469127

[B33] Zhuang L Lin J Lu ML Solomon KR Freeman MR Cholesterol-rich lipid rafts mediate akt-regulated survival in prostate cancer cells Cancer Res 2002 62 2227 2231 11956073

[B34] Boudreau DM Yu O Buist DS Miglioretti DL Statin use and prostate cancer risk in a large population-based setting Canc Causes Contr 2008 19 767 774 10.1007/s10552-008-9139-4 PMC2575006 18322813

[B35] Browning DR Martin RM Statins and risk of cancer: a systematic review and metaanalysis Int J Cancer 2007 120 833 843 10.1002/ijc.22366 17131313

[B36] Dale KM Coleman CI Henyan NN Kluger J White CM Statins and cancer risk: a meta-analysis JAMA 2006 295 74 80 10.1001/jama.295.1.74 16391219

[B37] Green J Cairns BJ Casabonne D Wright FL Reeves G Beral V Height and cancer incidence in the Million Women Study: prospective cohort, and meta-analysis of prospective studies of height and total cancer risk Lancet Oncol 2011 12 785 794 10.1016/S1470-2045(11)70154-1 21782509 PMC3148429

[B38] Renehan AG Height and cancer: consistent links, but mechanisms unclear Lancet Oncol 2011 12 716 717 10.1016/S1470-2045(11)70193-0 21782510

[B39] Schouten LJ Rivera C Hunter DJ Spiegelman D Adami HO Arslan A Height, body mass index, and ovarian cancer: a pooled analysis of 12 cohort studies Cancer Epidemiol Biomarkers Prev 2008 17 902 912 10.1158/1055-9965.EPI-07-2524 18381473 PMC2572258

[B40] Zuccolo L Harris R Gunnell D Oliver S Lane JA Davis M Height and prostate cancer risk: a large nested case-control study (ProtecT) and meta-analysis Cancer Epidemiol Biomarkers Prev 2008 17 2325 2336 10.1158/1055-9965.EPI-08-0342 18768501 PMC2566735

[B41] Neaton JD Kuller LH Wentworth D Borhani NO Total and cardiovascular mortality in relation to cigarette smoking, serum cholesterol concentration, and diastolic blood pressure among black and white males followed up for five years Am Heart J 1984 108 759 769 10.1016/0002-8703(84)90669-0 6475745

[B42] Strandberg TE Inverse relation between height and cardiovascular mortality in men during 30-year follow-up Am J Cardiol 1997 80 349 350 10.1016/S0002-9149(97)00362-7 9264435

[B43] Turley ML Tobias M Lawes CM Stefanogiannis N Vander HS Mhurchu CN Cardiovascular mortality attributable to high blood cholesterol in New Zealand Aust N Z J Public Health 2006 30 252 257 10.1111/j.1467-842X.2006.tb00866.x 16800202

